# Effects of genetic deletion of the Kv4.2 voltage-gated potassium channel on murine anxiety-, fear- and stress-related behaviors

**DOI:** 10.1186/2045-5380-2-5

**Published:** 2012-03-02

**Authors:** Carly Kiselycznyk, Dax A Hoffman, Andrew Holmes

**Affiliations:** 1 Laboratory of Behavioral and Genomic Neuroscience, National Institute on Alcohol Abuse and Alcoholism, NIH, Bethesda, MD, USA; 2 Molecular Neurophysiology and Biophysics Unit, Laboratory of Cellular and Synaptic Neurophysiology, Eunice Kennedy Shriver National Institute of Child Health and Human Development, NIH, Bethesda, MD, USA

## Abstract

**Background:**

Potassium channels have been proposed to play a role in mechanisms of neural plasticity, and the Kv4.2 subunit has been implicated in the regulation of action-potential back-propagation to the dendrites. Alterations in mechanisms of plasticity have been further proposed to underlie various psychiatric disorders, but the role of Kv4.2 in anxiety or depression is not well understood.

**Methods:**

In this paper, we analyzed the phenotype Kv4.2 knockout mice based on their neurological function, on a battery of behaviors including those related to anxiety and depression, and on plasticity-related learning tasks.

**Results:**

We found a novelty-induced hyperactive phenotype in knockout mice, and these mice also displayed increased reactivity to novel stimulus such as an auditory tone. No clear anxiety- or depression-related phenotype was observed, nor any alterations in learning/plasticity-based paradigms.

**Conclusions:**

We did not find clear evidence for an involvement of Kv4.2 in neuropsychiatric or plasticity-related phenotypes, but there was support for a role in Kv4.2 in dampening excitatory responses to novel stimuli.

## Background

The excitability and functional plasticity of specific brain circuits in cortical, limbic, and midbrain regions is thought to mediate behavioral responses to environmental threats, and to enable adaptation to stressors. At the cellular level, voltage- and calcium-activated potassium (K+) channels provide an important means of modulating neuronal activity and synaptic plasticity. Their function is further refined via the presence of multiple Kv primary and auxiliary subunits that differ in their voltage-dependence, post-translational regulation, sub-cellular localization and regional pattern of expression in the brain to accomplish distinct physiological functions.

A-type K+ channels composed of Kv4 subunits transmit a rapidly activating and inactivating current [1]. Kv4.2 mRNA is expressed in the periphery and in various brain regions involved in mediating stress-related behaviors, including the medial prefrontal cortex (mPFC), hippocampus and hypothalamus [2]. In CA1 hippocampal neurons, Kv4.2 protein is primarily localized on dendrites [3] where they exert their strongest functional effects [4]. Gene deletion of Kv4.2 (*Kcnd2*) in mice eliminates most of the A-type K+ current in hippocampal and cortical neurons [5-7], and increases back-propagation of the action potential from the axon to the somatodendritic region [6]. These and other effects of Kv4.2 knockout (KO) may be countered to some extent by compensatory increases in the expression of other Kv subunits [8], and increased inhibitory transmission in the hippocampus [9]. Notwithstanding, loss of Kv4.2 function produces a net increase in neuronal, and particularly dendritic, hyperexcitability in the mPFC and hippocampus, and possibly other brain regions in which this subunit is expressed.

Synaptic plasticity is also altered by Kv4.2 deletion, as evidenced by the enhanced CA1 hippocampal long-term potentiation (LTP) and increased threshold for long-term depression (LTD) seen in Kv4.2 (KO) mice [6,10]. Further demonstrating an important contribution to hippocampal plasticity, Kv4.2 channels are internalized during LTP induction, and Kv4.2 overexpression or deletion alters synaptic expression of N-Methyl-D-aspartate (NMDA) receptor subunits (GluN2A, GluN2B) to respectively constrain or promote hippocampal LTP [11,12]. Conversely, NMDAR throughput decreases hippocampal Kv4.2 through degradation [13,14] whereas concurrently causing an increase in Kv4.2 translation [15]. The reciprocal relationship between Kv4.2 and NMDA receptor subunits is particularly intriguing in the context of prior studies showing that gene KO of GluN2A [16] or GluN2B [17] disrupts anxiety-related behaviors and adaptations to stress.

Behaviorally, Kv4.2 KO mice are viable and appear grossly normal [18]. However, Kv4.2 KO mice show augmented nociceptive responses to thermal and mechanical stimulation [8] and increased sensitivity to the pro-convulsive effects of kainate, with no apparent increase in spontaneous seizures [19]. Furthermore, in a recent study assessing Kv4.2 KO mice on a 129S6/SvEvTac (129S6) background on a range of behavioral assays, Lockridge *et al. *found that the KO mice had reduced grip strength on a wire-hang test and increased locomotor activity in a brightly illuminated open field, but not in a dimly illuminated one [20]. Although the KO mice displayed an increased tendency to enter into the aversive central area of the open field, consistent with an anxiolytic-like phenotype, the mutants were no different from the wild-type (WT) controls in the increased plus-maze test for anxiety-such as behavior. This study also found that Kv4.2 KO mice showed increased immobility in the forced-swim test (FST) (but not the tail-suspension test) for 'depression-related' behavior, and were insensitive to the anti-immobility effects of the antidepressant fluoxetine, but not imipramine or desipramine, in the FST. In addition, *ex vivo *slice physiological recordings indicated that a form of 5-hydroxytryptamine (5-HT)2A receptor-mediated excitatory synaptic transmission in mPFC pyramidal neurons was attenuated in Kv4.2 KO mice after a single forced swim exposure, but only after repeated exposures in WT controls. Based on these findings, Lockridge *et al. *concluded that Kv4.2 deletion produces abnormal behavioral responses to stress, and suggested that this might be related to excessive 5-HT release.

The aim of the present study was to confirm and extend the characterization of Kv4.2 KO mice in stress- and other 'emotion'-related phenotypes. KO mice and WT littermate controls were compared for a battery of sensory and neurological tests (including hot-plate nociception, acoustic startle and prepulse inhibition of startle, and home-cage locomotion), exploratory and anxiety-related behaviors (novel open field, light/dark exploration, elevated plus-maze tests), pavlovian fear conditioning and extinction, and 'depression-related' behavioral responses (single and repeated inescapable forced-swim exposure). Phenotyping was conducted after the mutants were backcrossed onto a C57BL/6J background strain. This is important because genetic background can strongly influence the phenotype of mutant mice [21,22], and the aforementioned studies by Lockridge and colleagues tested the KO mice on a different genetic background (129S6).

## Methods

Experimental procedures were performed in accordance with the NIH Guide for Care and Use of Laboratory Animals and were approved by the local Animal Care and Use Committee.

### Animals

Kv4.2 KO mice were generated in a 129S6 genetic background as described previously [19]. For the current experiments, mice were backcrossed to the C57BL/6J strain for nine generations. To control for potential effects of maternal genotype [22], KO mice and WT controls were littermates bred from heterozygous parents. Males and females were used. Mice were housed in same-sex groupings in a temperature- and humidity-controlled vivarium under a 12-hour light/dark cycle (lights on at 06.00 hours) and testing conducted during the light phase after 1 hour of acclimation to the test room. Three separate, naïve cohorts were tested. One cohort of mice was screened on a battery of neurological measures, including prepulse inhibition and home-cage activity, and basal home-cage corticosterone levels. A second cohort was tested on a battery of assays for anxiety-, fear- and stress-related phenotypes, with putatively more stressful tests conducted later in the sequence and at least 6 days between each test: novel open field, light/dark exploration, elevated plus-maze, pavlovian fear conditioning and extinction, and forced-swim test/swim-induced corticosterone response. A third cohort was tested for behavioral responses to repeated inescapable forced swims. The experimenter remained blinded to genotype during testing. The number of mice tested in each assay is given in the corresponding figure legends.

### Initial phenotypic screen

#### Neurological test battery

##### Functional observational battery

Gross physical and neurological abnormalities were examined using a simple functional observation battery, as described previously [16,23,24]. Basic physical health was evaluated by examining for missing whiskers, bald patches, piloerection, exophthalmus, straub tail, kinked tail, kyphosis, lordosis, core body temperature, reactivity to handling, and body weight. Simple sensory reflexes were measured via orienting responses to an approaching hand and to physical touch via palpebral closure on touch of the eye, twitch of pinna on touch, and an orienting response to tail pinch. Mice were observed for splayed limbs, and forepaw and hind limb clutch when suspended upside-down by the tail, and wild running, freezing, trembling, sniffing, licking, rearing, jumping, seizures, defecation, urination, head bobbing, circling, abnormal gait, retropulsion, and prancing forelimbs when placed in a novel, bare, standard holding cage (130 × 135 × 350 mm) for 1 minute.

##### Nociception

Pain perception was tested using the hot-plate test. a flat plate (Columbus Instruments, Columbus, OH, USA) was heated to 55°C, and the mouse placed upon it. The latency to first hind-paw lick was manually timed, with a maximum response latency of 30 seconds. and genotypes statistically compared via Student's t-test.

##### Acoustic startle and prepulse inhibition of startle

Acoustic startle and prepulse inhibition of startle (PPI) was tested as described previously [25]. The mouse was placed in a clear Plexiglas holding cylinder (San Diego Instruments SR-LAB System; San Diego, CA, USA) for an acclimation period of 5 minutes. A 65-dB broadband background noise was presented during acclimation and throughout the test session. Five pulse-alone trials (120 dB broadband sound pulse for 40 ms) began and ended the session, and were not included in the analysis. Testing consisted of presentation of 120 dB startle trials and prepulse + startle trials (noise prepulse sound for 20 ms, followed 100 ms later by the 40 ms 120-dB broadband sound pulse). There were three different prepulse intensities (3, 6 and 12 dB above background), each presented 10 times with a variable interval (range 12-30 seconds) between each presentation. There were vie pulse-alone trials before and after each prepulse + startle trial. Basal activity in the startle chambers was measured during the no-stimulus trials. Startle amplitude was measured starting from the onset of the startle stimulus via whole-body vibrations (sampled every 1 ms over 65 ms), transduced into analog signals by a piezoelectric unit attached to the platform on which the cylinders rested. This was used to derive an average startle amplitude over the 65 ms recording period. PPI at each prepulse intensity was calculated as:

$$100-\frac{\text{startle}\;\text{response}\;\text{for}\;\text{prepulse}+\text{startle}\;\text{trials}}{\text{startle}\;\text{response}\;\text{for}\;\text{startle}-\text{alone}\;\text{trials}}\times 100$$

The effects of genotype and PPI intensity were analyzed using two-way analysis of variance (ANOVA), with repeated measures for PPI intensity.

##### Home-cage activity

The mouse was individually housed in a standard holding cage as before, and left undisturbed for a 48-hour acclimation period under normal vivarium conditions. Activity was measured over 24 hours using a photocell-based activity monitor (Opto M3; Columbus Instruments, Columbus, OH, USA) and expressed as the 12-hour average activity during the light and dark phase, as described previously [26]. The effects of genotype and circadian phase were analyzed using two-way ANOVA, with repeated measures for cycle.

### Battery of anxiety-, fear-, and stress-related behaviors

#### Anxiety-related behaviors

##### Novel open field

The novel open-field test [27] was conducted as described previously [28] in a 400 × 400 × 350 mm square arena (60 lux) constructed of white Plexiglas. Testing was conducted under 65 dB white noise to minimize external noise disturbances (Sound Screen, Marpac Corporation, Rocky Point, NC, USA). The mouse was placed in the perimeter and allowed to explore the apparatus for 30 minutes. Total distance traveled and time spent in the center square (200 × 200 mm) was measured by a video-tracking system (Ethovision; Noldus Information Technology Inc., Leesburg, VA, USA).

##### Light-dark exploration test

The light-dark exploration test [24] was conducted as described previously [29]. The mouse began the test in an opaque black Plexiglas shelter (390 × 130 × 160 mm) with a 130 × 80 mm opening at floor level that opened onto a large white Plexiglas square arena (390 × 390 × 350 mm) illuminated to approximately 90 lux. Percentage time spent in the lighted compartment, frequency of entering the lighted compartment, and total distance traveled in the entire apparatus over a 15 min session were measured by a video-tracking system (Ethovision Noldus Information Technology Inc). Mice that did not enter the lighted compartment were recorded as having a latency to exit time of 900 seconds. Genotypes were compared using Student's t-test.

##### Elevated plus-maze

The elevated plus-maze test [30] was conducted as described previously [31]. The apparatus consisted of two open arms (300 × 50 mm; 90 lux) and two closed arms (300 × 50 × 150 mm; 20 lux), extending from a 50 × 50 mm central area and elevated 200 mm from the ground (San Diego Instruments, San Diego, CA, USA). The walls were made from black acrylonitrile butadiene styrene (ABS) plastic and the floor from white ABS plastic. A 50 mm high raised lip around the perimeter of the open arms prevented the mice from falling off the maze. Testing was conducted under 65 dB white noise (Sound Screen, Marpac Corporation, Rocky Point, NC, USA) to minimize external noise disturbance. The mouse was placed in the center of the maze facing an open arm, and allowed to explore the apparatus for 6 minutes. Time spent in the open arms and number of entries into the open and closed arms were measured by a video-tracking system (Ethovision Noldus Information Technology Inc). Genotypes were compared using Student's t-test.

#### Fear-related behaviors

##### Pavlovian fear conditioning and extinction

Pavlovian fear conditioning and extinction was assessed as described previously [32,33]. The mouse was placed in context A: a 270 × 270 × 110 mm chamber with transparent walls and a metal rod floor. To provide a distinctive olfactory environment, the chamber was cleaned between subjects with a 79% ethanol/20% water/1% vanilla extract solution. After acclimation period of 180 seconds, the mouse received three pairings (interval of 60-120 seconds after each pairing) of an auditory tone (30 seconds, 80 dB, white noise) and footshock (2 seconds, 0.6 mA scrambled footshock), in which the shock was presented during the last 2 seconds of the tone. The presentation of stimuli was controlled by a freeze monitoring system (Med Associates Inc, Georgia, VT, USA).

Twenty-four hours later, expression of fear to the tone and subsequent within-session extinction was tested. Mice were placed in context B: a novel context (Plexiglas cylinder with black/white-checkered walls and a solid floor, cleaned with a 1% acetic acid/99% water solution) housed in a novel room. After an initial acclimation period of 180 seconds, the mouse received 500 presentations of the tone alone (each tone for 30 seconds, with a no-stimulus interval of 5 seconds). Twenty-four hours later, extinction retrieval was probed with three tone presentations in context A. Four hours later, mice were presented with three tones in context B to assess fear renewal.

Freezing (no visible movement except that required for respiration) in response to the tone was manually scored every 5 seconds and converted to a percentage:

(numberoffreezingobservations/totalnumberofobservations×100).

Freezing during extinction trials were averaged into 100 five-trial blocks for analysis, and the first and last trial blocks compared. The effect of genotype and trial/trial block during conditioning and extinction were analyzed using two-factor ANOVA, with repeated measures for trial/trial block, followed by Bonferroni *post hoc *tests. The effect of genotype on extinction retrieval and fear renewal were analyzed using Student's t-test.

#### Responses to swim stressors

##### Forced-swim test behavior and corticosterone response

The forced-swim test [34] was conducted as described previously [35]. The mouse was gently lowered into a 200 mm-diameter cylinder filled with water 24 ± 1.0°C for test lasting 6 minutes. Immobility (cessation of limb movements except for minor movement necessary to keep the mouse afloat) was manually scored every 5 seconds during minute 3 to 6. Genotypes were compared using Student's t-test.

Mice were returned to the home cage for 30 minutes after forced swim, and then killed via cervical dislocation and decapitated. The trunk blood was collected, left to coagulate at room temperature for 1-2 hours, and then separated by centrifugation at 4°C for 30 seconds at 13,000 rpm (20000 x g). Serum was collected and analyzed for corticosterone (bound and free) using a commercial radioimmunoassay (MP Biomedicals, Orangeburg, NY) as described previously [36]. Genotypes were compared using Student's t-test.

##### Repeated inescapable forced swim

The repeated inescapable forced-swim (riFS) test was conducted as described previously [17]. The mouse was gently lowered into a cylinder filled with water at 24 ± 1.0°C. The cylinder was of a larger diameter (300 mm) than that used for the forced-swim test used above (200 mm) and had a 40 × 40 mm escape hole located 40 mm above the waterline and out of reach for the mouse. A platform (plastic wiffle ball) was magnetically held 100 mm below the water, also out of reach. After 1 minute, the platform was remotely released (by turning off the magnet) and quickly (within < 2 seconds) floated vertically upwards along a pole to sit on top of the water, directly under the escape hole. If the mouse attempted to climb onto or hold the platform, it sank below the water. The test terminated 20 seconds after the platform was presented, regardless of the number of attempts by the mouse to use it to reach the escape hole. There was one trial per day (10.00 to 12.00 hours) for 10 consecutive days. Immobility was manually scored every 5 seconds during the 1 minute before platform presentation. The effect of genotype and trial were analyzed using two-way ANOVA, with repeated measures for trial.

### Statistical analysis

Knockout and wildtype mice were compared using Student's t-test with the exception of the prepulse inhibition of startle, home cage activity, fear conditioning and extinction, and riFS testing, which was analyzed using a 2-factor analysis of variance (ANOVA) with Bonferroni posttest. The threshold for statistical significance was *P *< .05. GraphPad Prism 5 statistical software (La Jolla, CA) was used for all statistical analysis.

## Results

### Normal gross behavior and neurological functions

**Figure 1:**
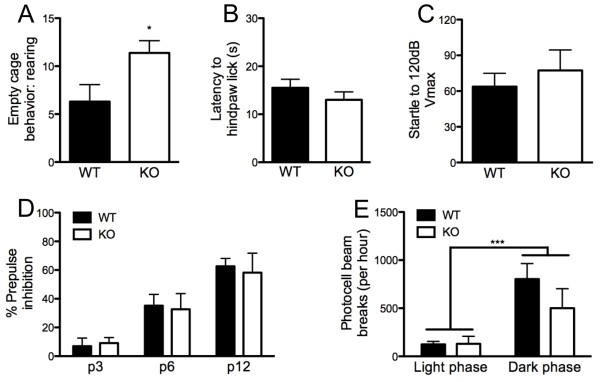
**Increased exploratory rearing in Kv4.2 knockout (KO) mice**. **(A) **KO mice showed more rearing behavior than wild-type (WT) controls in a novel empty-cage environment. **(B) **Genotypes did not differ in nociception as measured by the hot-plate tes, or in **(C) **the acoustic startle response **(D) **or prepulse inhibition of startle. **(E) **Light and dark phase home-cage activity was similar between genotypes. Data are mean ± SEM; n = 8-10**/**genotype. *P < 0.05; **P < 0.01.

The different genotypes did not differ on most measures of physical health, gross behavior, or neurological functions (Table 1), with the exception of increased rearing seen in KO mice relative to WT controls when placed in an empty cage for 1 min (t_(16) _= 2.21, *P *< 0.05) (Figure 1A). Hot-plate nociception was not different between genotypes (Figure 1B). KO mice also showed no alterations in acoustic startle compared with WT controls (Figure 1C). There was a significant effect of prepulse intensity (*F*_(2,32) _= 35.65, *P *< 0.01) but not of genotype, and no intensity × genotype interaction for PPI (Figure 1D). There was a significant effect of circadian phase for home-cage activity (*F*_(1,15) _= 23.94, *P *< 0.01), with both genotypes showing more activity during the dark phase than the light phase (Figure 1E) (after one mouse per genotype with outlying scores (> 2 SD) were excluded). There was no effect of genotype or phase × genotype interaction for home-cage activity.

**Table 1:** Empty-cage behaviors, physical health, sensory reflexes, and neurological functions

Empty-cage behaviors^a^	WT^b^	KO^c^
Freezing	0	0
Trembling	0	0
Sniffing	100	100
Licking	0	0
Rearing	80	100
Jumping	0	0
Seizure	0	0
Defecation	60	25
Urination	0	0
Head bobbing	0	0
Circling	0	0
Abnormal gait	0	0
Retropulsion	0	0
Physical health		
Missing whiskers	0	0
Bald patches	0	0
Exopthalmus	0	0
Straub tail	0	0
Kinked tail	0	0
Kyphosis	0	0
Lordosis	0	0
Body weight, g^e^	23.6 ± 0.8	23.9 ± 1.3
Core body temp, °C^e^	36.2 ± 0.3	36.8 ± 0.1
Sensory reflexes		
Approach response	100	100
Touch response	100	100
Palpebral response	100	100
Pinna reflex	100	100
Tail pinch response	100	100
Motor, neurological		
Splayed limbs	100	100
Forepaw clutch	0	0
Hind-paw clutch	0	0

^a^Data denote the percentage of animals showing a response unless specified otherwise,

^b^Wild-type.

^c^Knockout.

^e^Mean ± SD.

### Increased exploratory locomotion and test-specific decreased anxiety-like behavior

**Figure 2:**
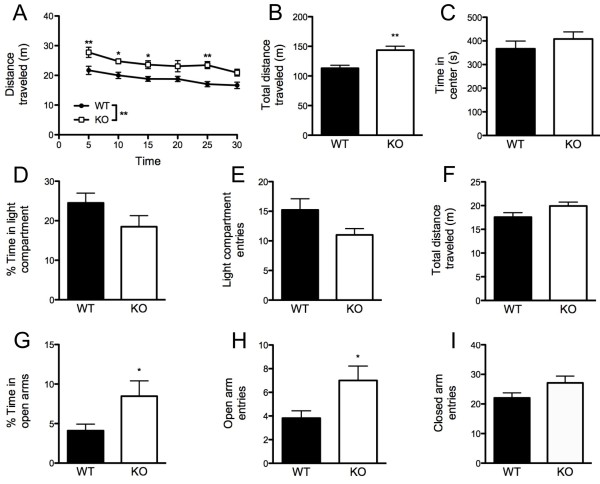
**Increased open-field locomotor activity and test-specific decreases in anxiety-like behavior in Kv4.2 knockout (KO) mice**. KO mice traveled farther than wild-type (WT) controls in a novel open-field test, as shown via **(A) **a time-course analysis) and **(B) **over the full session. **(C) **Time spent in the center of the open field did not differ between genotypes. In the light/dark exploration test, genotypes did not differ in **(D) **percentage time spent in the lighted compartment, **(E) **entries into the lighted compartment or **(F) **distance traveled throughout the apparatus. In the elevated plus maze, KO mice spent a greater percentage of the session **(G) **in the open arms and made more entries into **(H) **the open arms, but not into **(I) **the closed arms, compared with wild-type (WT) controls. Data are mean ± SEM; n = 8-12**/**genotype. *P < 0.05; **P < 0.01.

In the novel open-field test, time-course analysis revealed a significant effect of genotype (*F*_(1,18) _= 13.70, *P *< 0.01) and time bin (*F*_(5,90) _= 14.13, *P *< 0.01), but no interaction for distance traveled (Figure 2A). KO mice traveled significantly farther than WT controls over the whole session (t_(18) _= 3.70, *P *< 0.01) (Figure 2B). Genotypes did not differ in the time spent in the center of the open field (Figure 2C).

In the light/dark exploration test, genotypes did not differ in percentage time (Figure 2D) or entries into (Figure 2E) the lighted compartment during the first or last (data not shown) 5 minutes. Genotypes also did not differ in distance traveled in both compartments during the first (data not shown) or last 5 minutes of the session (Figure 2F).

In the elevated plus maze, KO mice spent significantly more time in the open arms (t_(17) _= 2.30, *P *< 0.05) (Figure 2G), and made significantly more entries into the open arms (t_(17) _= 2.52, *P *< 0.05) (Figure 2H), compared with WT controls. Genotypes did not differ in the number of entries into the closed arms (Figure 2I). One WT mouse with outlying (> 2 SD) was excluded from each of the three elevated plus maze measures (time in open arms, open-arm entries, closed-arm entries).

### Increased freezing to unconditioned auditory tones

**Figure 3:**
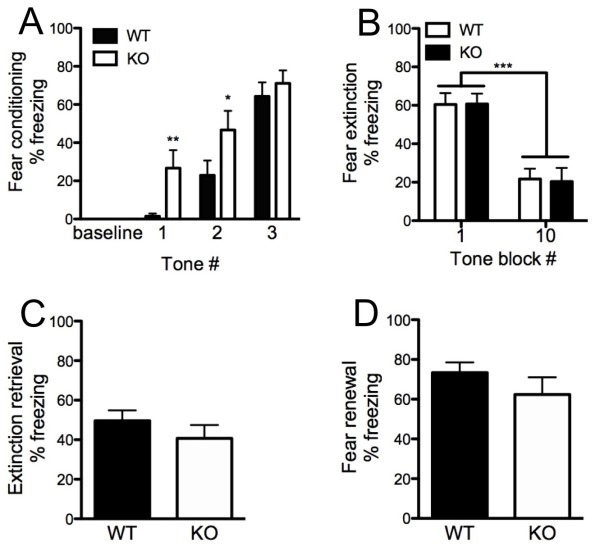
**Increased freezing to unconditioned tones in Kv4.2 knockout (KO) mice**. **(A) **During conditioning KO mice froze more than wild-type (WT) controls during the first (before conditioning) and second conditioned stimulus (CS) presentation trials. **(B) **Genotypes showed equivalent initial freezing and similar reductions in freezing from the first to the last five CS trial block during extinction training. Genotypes did not differ in freezing during **(C) **extinction retrieval or **(D) **fear renewal. Data are mean ± SEM; n = 8-12**/**genotype. *P < 0.05; **P < 0.01.

During conditioning, there was significant interaction between the conditioned stimulus (CS) trial and genotype (*F*_(1,21) _= 6.28, *P *< 0.05) for freezing to the CS tone. *Post hoc *tests revealed higher freezing to the first (that is, before any foot shock) and second CS trial but not to the third CS trial in KO mice (Figure 3A).

During extinction training, there was a significant effect of trial block (*F*_(9,189) _= 15.25, *P *< 0.01), and significant reduction in freezing to the last five tones compared with the first five (*F*_(1,21) _= 61.35, *P *< 0.01). However, there was no effect of genotype or trial block × genotype interaction for freezing (Figure 3B).

Genotypes did not differ in freezing to the CS during either the extinction-retrieval (Figure 3C) or context-renewal tests (Figure 3D). Freezing during the baseline pre-CS periods was negligible, and was not different between genotypes for extinction training (WT = 9.5 ± 2.4, KO = 6.4 ± 1.4) or retrieval (WT = 4.2 ± 1.2, KO = 1.2 ± 1.2).

### Exaggerated swim-induced corticosterone and normal 'depression-related' responses to single and repeated forced swim

**Figure 4:**
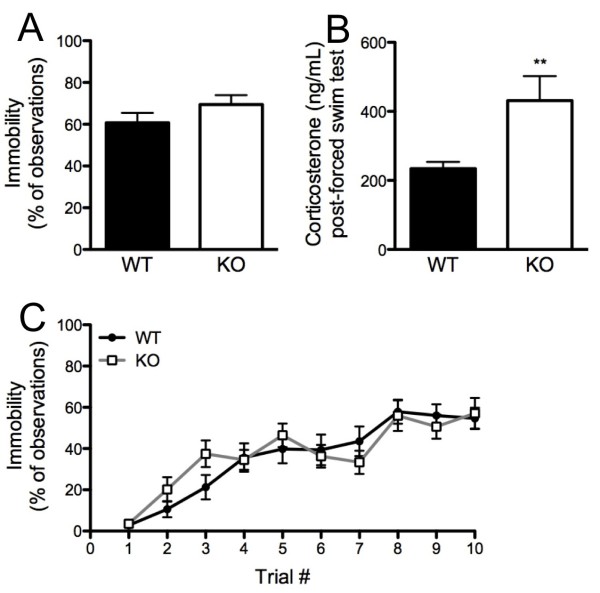
**Exaggerated swim-induced corticosterone and normal responses to swim stressors in Kv4.2 knockout (KO) mice**. **(A) **Genotypes did not differ in percentage immobility during a single 6-minute forced-swim exposure (n = 9-13/genotype). **(B) **KO mice had significantly raised serum corticosterone levels after the single forced-swim exposure (n = 9-13/genotype). **(C) **Genotypes had a similar increase in percentage immobility increased over 10 × 1-min inescapable forced swim exposures (n = 14-18**/**genotype). Data are mean ± SEM. **P < 0.01.

During a single forced-swim exposure, genotypes did not differ in percentage of time spent immobile (Figure 4A). However, serum corticosterone levels after this swim exposure were significantly higher in KO mice than WT controls (t_(19) _= 3.27, *P *< 0.01) (Figure 4B). Based on this genotype difference, a separate cohort of stress-naïve mice was killed immediately on removal from the cage for corticosterone analysis. Corticosterone levels did not differ between genotypes under these non-stressed conditions (WT = 40.4 ± 4.3 mg/mL, KO = 109.5 ± 41.8 mg/mL). Three WT and one KO sample were removed because of errors in the blood-collection assay.

On the riFS test, there was a significant effect of trial (*F*_(9,270)__ = 27.85, *P *< 0.01) but not of genotype, and no trial × genotype interaction for percentage immobility (Figure 4D).

## Discussion

The aim of the current study was to examine the effects of deleting the A-type voltage-gated K+ channel Kv4.2 on multiple stress- and other 'emotion'-related phenotypes. We found a complex set of significant phenotypic alterations in Kv4.2 mice. These included increased exploratory activity in novel environments, test-specific decreases in anxiety-like behavior, increased fear response to auditory stimuli, and an exaggerated corticosterone response to stress.

Consistent with previous studies [19], an initial observational battery showed no gross alterations in various measures of health or sensory function in the Kv4.2 knockout; however, this analysis did reveal increased rearing in the KO mice, suggestive of heightened levels of exploratory behavior. Similarly, exploratory locomotion in a novel open field was robustly increased in the KO mice, at least over a 30 minute session (longer sessions were not examined). These genotype effects did not seem to reflect generalized locomotor hyperactivity in the mutants, as evidenced by normal levels of activity in the familiar and low-stress environment of the home cage. Rather, these data suggest that Kv4.2 deletion produced an augmented behavioral reaction to novel and/or stressful environments.

This behavioral phenotype may be related to the neural abnormalities that have been reported in Kv4.2 KO mice. For example, these mice have been found to exhibit neuronal and dendritic hyperexcitability due to increased axonal back-propagation [6]. Interestingly in this context, we have previously identified a similar profile in the same behavioral assays after deletions of other genes involved in regulating neuronal excitability, such as components of the glutamate signaling system [16,37,38]. This overlap indicates that genetic loss of molecules involved in regulating neuronal excitability and synaptic plasticity produces some convergent phenotypic effects on novelty-driven behavioral reactivity. Although hyperlocomotion in a novel environment is often taken as a measure relevant to schizophrenia [39,40], the Kv4.2 KO mice in the present study did not show changes in another schizophrenia-relevant measure, prepulse inhibition of startle, unlike our findings in previous studies of glutamate deletions. Therefore, without additional experiments to interrogate this issue further, it would be premature discuss the Kv4.2 KO phenotype in terms of potential relevance to that disease.

Novelty-induced hyperactivity can confound interpretation of behavior in tests for anxiety-like behavior due to non-specific increases in activity [41]. However, we found that Kv4.2 KO mice were no different from WT controls in the light/dark exploration test and showed increased open-arm exploration in the elevated plus maze, which was not associated with increased general locomotor activity in this test (as measured by the number of closed-arm entries). This suggests a test-specific anxiolytic-like phenotype in the KO mice, albeit with the caveat that the classic conflict-based assays for anxiety-like behavior cannot unequivocally parse an anxiety-like decrease in avoidance from a novelty-driven increase in approach [41]. Notwithstanding, the test-specific nature of this phenotype is notable, and echoes earlier studies showing that the elevated plus maze can be particularly sensitive to certain gene mutations, possibly because it is inherently more stressful than ostensibly similar tests such as the light/dark exploration [42]. This would generally fit with a profile of exaggerated behavioral responses of Kv4.2 KO mice, particularly under conditions of strong environmental 'provocation.'

Consistent with this interpretation, the KO mice showed increased unconditioned freezing to an auditory stimulus, and an augmented corticosterone response to a single exposure to the forced-swim test. By contrast, the behavioral response (immobility) to forced swim, a very stressful and 'provocative' situation, was normal in the KO mice. Increases in immobility produced by repeated, brief, forced-swim exposures were also similar between KO mice and WT controls. Thus, increased behavioral reactivity in the mutants does not generalize to altered immobility during various forms of forced-swim exposure. One explanation is enhanced tonic inhibitory transmission [9] served to mitigate the penetrance of the phenotype under forced-swim conditions.

The absence of changes in forced-swim tests in the current study differs from the previous finding by Lockridge *et al. *that these mice showed increased immobility in the forced-swim test [20]. Indeed, whereas the earlier report found, as we did, novel open-field hyperactivity, some other differences were seen in the earlier study, including no change in elevated plus maze anxiety-like behavior. Other than methodological variations, the principal salient factor potentially explaining these apparent discrepancies is genetic background. Kv4.2 KO mice were bred on a C57BL/6J background for the current study and a 129S6 background for the previous study. Genetic background can have a profound influence on the penetrance and expression of emotion-related phenotypes in mutant mice, because of epistatic interactions between a mutation and modifier genes [21,22]. It seems likely that such interactions shaped the phenotypic profile of the Kv4.2 KO mice, and it would be interesting to explore this further by identifying the responsible modifiers.

Another important avenue for future studies will involve assessment of Kv4.2 KO mice for learning and memory. The mutants have enhanced hippocampal synaptic plasticity [6,10], which is associated with altered synaptic expression of synaptic proteins that are strongly implicated in learning by mutant studies [23,43-45], such as the NMDA receptor GluN2A and GluN2B subunits [11-13]. In the current study we found that the KO mice displayed normal amygdala-mediated fear learning and mPFC-mediated fear extinction. Given the localization of synaptic alterations to the hippocampus, it will be valuable to supplement these findings by assessing Kv4.2 KO mice for hippocampal-dependent forms of learning.

## Conclusions

In summary, we found that constitutive deletion of the A-type voltage-gated K+ channel Kv4.2 produced a behavioral phenotype principally characterized by increased behavioral reactivity to exposure to some but not all novel environmental situations. This largely confirms earlier evaluation of these mice, although some discrepancies were found, possibly due to the influence of genetic background. These behavioral abnormalities extend the finding that deletion of Kv4.2 leads to exaggerated neuronal excitability in cortical and hippocampal regions involved in emotion regulation. These data shed further light on the role of Kv4.2 in regulating neural and behavioral functions.

## List of abbreviations

5-HT: 5-hydroxytryptamine; ABS: acrylonitrile butadiene styrene; CS: conditioned stimulus; KO: knockout; LTD: long-term depression; LTP: long-term potentiation; mPFC: medial prefrontal cortex; NMDA: N-Methyl-D-aspartate; PPI: prepulse inhibition of startle; WT: wild-type.

## Competing interests

The authors declare that they have no competing interests.

## Authors' contributions

CK carried out the behavioral testing and drafted the manuscript, DH generated the Kv4.2 mice and aided in manuscript revision, and AH conceived of the study and helped draft the manuscript. All authors read and approved the final manuscript.
